# Safety recommendations for ALK tyrosine kinase inhibitors in non–small cell lung cancer: evidence from FAERS and CVARDD real-world databases

**DOI:** 10.3389/fonc.2026.1728791

**Published:** 2026-03-25

**Authors:** Yuanyuan Zhang, Tuanzhuang Zhang, Yuping Yang, Wenhui Zhang, Qiangping Ma, Juan Li, Jintian Li, Xuan Yang, Jirong Gong, Xinyi Wang, Jianqing Liang

**Affiliations:** 1 Clinical College of Traditional Chinese Medicine, Gansu University of Chinese Medicine, Lanzhou, China; 2 Orthopedics and Traumatology, Gansu Provincial Traditional Chinese Medicine Hospital, Lanzhou, China

**Keywords:** ALK tyrosine kinase inhibitors, hepatotoxicity, non–small cell lung cancer, pharmacovigilance, pleural effusion, sex differences

## Abstract

**Background:**

Non–small cell lung cancer (NSCLC) remains one of the leading causes of cancer mortality worldwide. The development of anaplastic lymphoma kinase tyrosine kinase inhibitors (ALK-TKIs) has significantly improved survival among patients with ALK-positive NSCLC. However, prolonged treatment and wider clinical use have led to increasing reports of adverse events (AEs). Existing studies have primarily explored individual drugs, with limited comparative evidence across different ALK-TKIs regarding sex-specific safety differences and time-to-onset patterns.

**Methods:**

This real-world pharmacovigilance study analyzed data from the U.S. Food and Drug Administration Adverse Event Reporting System (FAERS, Q1 2004–Q2 2025) and the Canadian Vigilance Adverse Reaction Database (CVARDD). Disproportionality analyses were performed using four algorithms—Reporting Odds Ratio (ROR), Proportional Reporting Ratio (PRR), Bayesian Confidence Propagation Neural Network (BCPNN), and Multi-Item Gamma Poisson Shrinker (MGPS)—to detect adverse event signals for crizotinib, alectinib, and brigatinib. Sex-stratified risk analyses, cross-database validation, and Weibull time-to-onset modeling were further conducted to assess robustness and temporal patterns of AE occurrence.

**Results:**

A total of 18–683 AE reports were identified (crizotinib = 9 030; alectinib = 8 486; brigatinib = 1 167). Distinct toxicity spectra were observed among the three ALK-TKIs. Brigatinib exhibited the strongest hepatotoxic and pulmonary signals, notably hepatic function abnormal (ROR = 13.95) and pleural effusion (ROR = 11.03), indicating a high risk of early liver and respiratory toxicity. Alectinib showed pronounced metabolic and edema-related AEs (oedema, ROR = 9.47; hepatic function abnormal, ROR = 9.21), suggesting a tendency toward fluid retention and hepatobiliary dysfunction. Crizotinib demonstrated a more balanced safety profile but still presented notable risks for pleural effusion (ROR = 8.88) and hepatic function abnormal (ROR = 7.92), both showing early-onset patterns (median TTO = 34.5 days and 14.5 days, respectively). Sex-stratified analyses revealed that males were more prone to renal, cardiac, and respiratory toxicities, whereas females were more likely to develop hepatic and hematologic events. Weibull modeling indicated an “early failure” pattern (β< 1) for all agents, meaning AEs predominantly occurred within the first 12 weeks of therapy. Cross-database validation confirmed consistent risk signal direction and strong reproducibility between FAERS and CVARDD datasets.

**Conclusions:**

All three ALK-TKIs demonstrate distinct, generation-dependent safety profiles characterized by early-onset hepatobiliary and pulmonary toxicities, with evident sex-specific differences in organ susceptibility. Intensive safety monitoring during the initial 12 weeks of therapy is essential for preventing severe outcomes.

## 1 Introduction

Non–small cell lung cancer (NSCLC) is one of the leading causes of cancer-related morbidity and mortality worldwide. Over the past two decades, substantial progress has been made in its treatment. The introduction of small-molecule tyrosine kinase inhibitors (TKIs) and immune checkpoint inhibitors has provided unprecedented survival benefits for selected patients. Nevertheless, the overall cure and survival rates of NSCLC remain unsatisfactory, particularly in metastatic disease (1, 2). Among the genetic drivers, rearrangements of the anaplastic lymphoma kinase (ALK) gene represent an important oncogenic alteration, accounting for approximately 3%–7% of all lung adenocarcinoma cases (3). Since the advent of ALK tyrosine kinase inhibitors (ALK-TKIs), both progression-free survival and overall survival have significantly improved, and ALK-TKIs have become the standard first-line therapy for ALK-positive NSCLC (4, 5). However, with longer treatment durations and broader real-world use, drug-related adverse drug events (ADEs) have increasingly attracted attention, highlighting the need for a deeper understanding of safety profiles and differential risks across commonly used ALK-TKIs.

In this study, we focused on crizotinib, alectinib, and brigatinib because they represent widely used agents spanning different generations of ALK inhibition and distinct therapeutic positioning in clinical practice (6). Crizotinib, as an earlier ALK inhibitor, historically served as a key first-line option and remains clinically relevant in certain settings; alectinib is a commonly used next-generation agent with established first-line use and improved central nervous system (CNS) disease control; brigatinib is also a next-generation inhibitor used for advanced ALK-positive NSCLC with activity against CNS metastases and resistance profiles (4–6). Importantly, these drugs differ in target spectrum, exposure profiles, and tissue distribution, which may translate into heterogeneous toxicity patterns. Prior studies and post-marketing evidence suggest that crizotinib is frequently associated with visual disturbances and hepatotoxicity, alectinib with constipation, weight gain, and edema, and brigatinib with hypertension, myalgia, and pulmonary toxicities (7–9). Because these agents are commonly considered as alternatives or sequential options in the treatment pathway, clarifying their comparative safety characteristics is clinically meaningful for individualized drug selection and monitoring strategies.

Beyond pharmacologic and structural differences among ALK-TKIs, patient-specific factors may also shape ADE susceptibility. Sex differences are increasingly recognized as determinants of drug response and toxicity, potentially mediated by differences in body composition, hormonal milieu, and pharmacokinetic processes such as absorption, distribution, metabolism, and elimination (4, 10). For example, sex-related variability in hepatic enzyme activity, transporter expression, and cardiovascular or metabolic vulnerability may contribute to differential toxicity manifestations across males and females (11, 12). However, existing literature has often emphasized efficacy endpoints or single-agent safety descriptions, whereas comparative, sex-stratified safety evaluations across multiple ALK-TKIs remain limited. This knowledge gap restricts evidence-based guidance for sex-informed risk assessment and targeted monitoring in routine practice.

Against this backdrop, real-world data (RWD) complement clinical trials by capturing broader populations, longer exposure periods, and rarer events. The U.S. Food and Drug Administration’s Adverse Event Reporting System (FAERS) and the Canadian Vigilance Adverse Reaction Database (CVARDD) collect large volumes of spontaneous adverse event reports from distinct healthcare systems and populations, enabling cross-database assessment of signal robustness (13, 14). Therefore, using FAERS and CVARDD, we systematically evaluated and compared the safety signal characteristics of crizotinib, alectinib, and brigatinib, and further examined sex differences, signal strength distributions, and time-to-onset patterns. By placing these agents within a comparative context aligned with their clinical use, our findings aim to clarify drug- and sex-specific safety discrepancies and provide practical evidence to support individualized risk management in ALK-positive NSCLC.

## 2 Materials and methods

### 2.1 Data source

The adverse drug event (ADE) data analyzed in this study were obtained from the U.S. Food and Drug Administration Adverse Event Reporting System (FAERS) database, which has been publicly accessible since 2004. FAERS collects spontaneous adverse event reports from diverse sources, including healthcare professionals, pharmaceutical manufacturers, and patients. To investigate ADEs associated with the ALK tyrosine kinase inhibitors (ALK-TKIs) crizotinib, alectinib, and brigatinib, all relevant reports were extracted from FAERS from its inception through the second quarter (Q2) of 2025. The data were subsequently imported into MySQL 15.0 and processed using Navicat Premium 15 software to facilitate comprehensive data management and analysis.

### 2.2 Data extraction and processing

In this study, crizotinib, alectinib, and brigatinib were identified as suspected drugs, and their names were standardized using the Medex_UIMA_1.8.3 encoding system. The extracted FAERS data were preprocessed using the Statistical Analysis System (SAS) and MySQL to ensure data completeness and integrity. Duplicate case reports with identical case identifiers in the DEMO table were removed. To address the inherent issue of duplicate reporting in spontaneous pharmacovigilance databases, a rigorous deduplication process was applied. In addition to removing reports with identical case identifiers in the DEMO table, we also cross-referenced reporting dates, patient demographics (age, sex), and reporting countries to identify and exclude overlapping or updated reports for the same patient event, retaining only the most recent and complete version.

The most recent version of the Medical Dictionary for Regulatory Activities (MedDRA version 27.1) was used to map the preferred terms (PTs) and corresponding system organ classes (SOCs) for ADEs associated with the three ALK-TKIs. Clinical characteristics of patients reporting ADEs related to these drugs were collected, including age, sex, weight, reporting country, indication, and reporting year.

**Figure 1:**
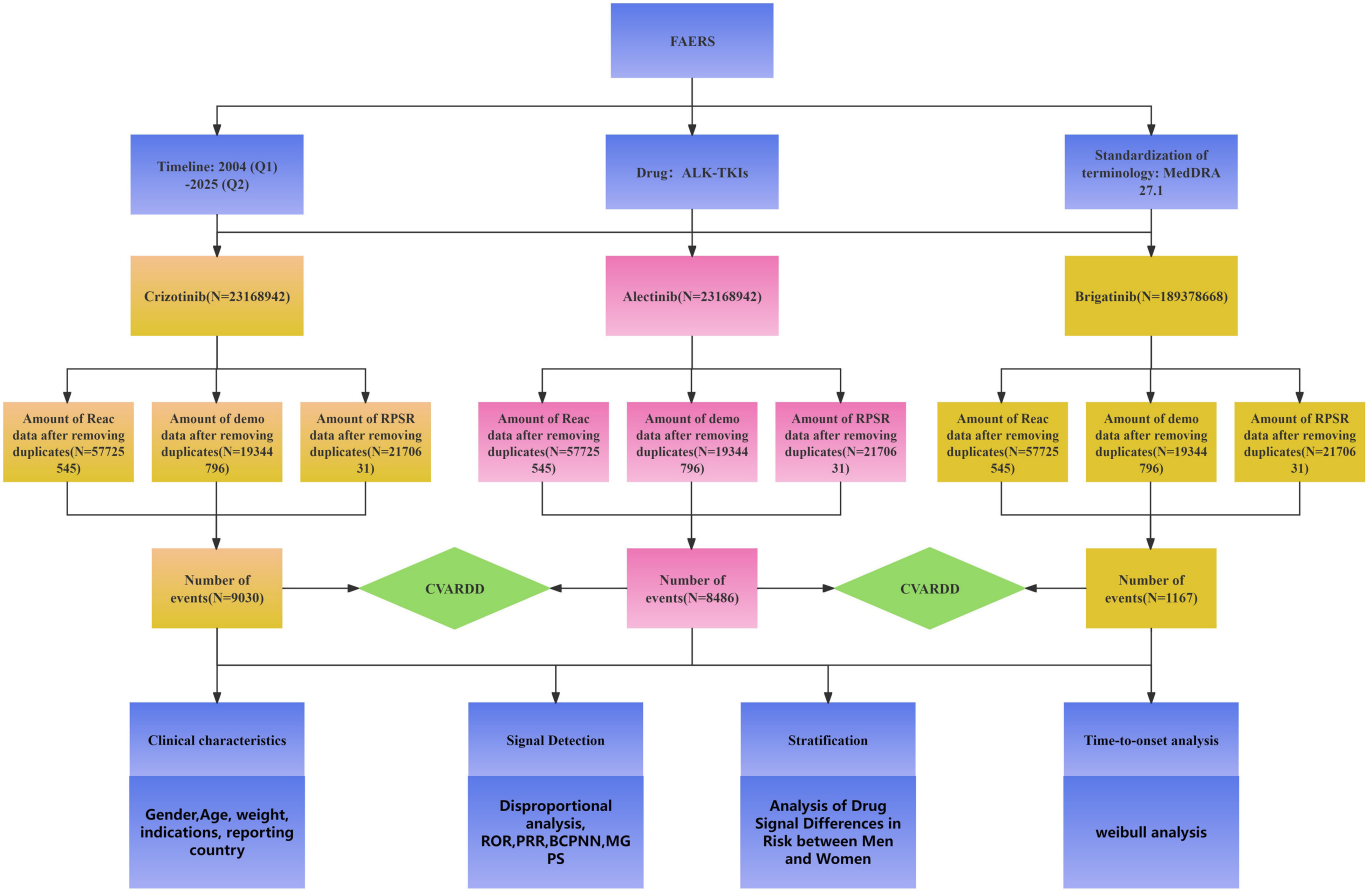
Workflow for the analysis of adverse events associated with ALK-TKIs (crizotinib, alectinib, and brigatinib).

The overall workflow for data extraction, preprocessing, and analysis of ALK-TKI–related adverse events is illustrated in **Figure 1**.

### 2.3 Data mining algorithms

To investigate the potential associations between ALK-TKI drugs (Crizotinib, Alectinib, Brigatinib) and Adverse Drug Events (ADEs), an disproportionality analysis was conducted. This analysis is considered a key tool in pharmacovigilance and aims to assess the relationship between drugs and ADEs by comparing observed frequency ratios in the exposed and unexposed populations using 2 × 2 contingency tables. This method is a cornerstone of pharmacovigilance, assessing the strength of the statistical association between a drug and an adverse event by comparing the observed frequency ratios in the exposed versus the unexposed populations. The study design and reporting of signal detection followed the Adverse Event Proportion Analysis Reporting (READUS-PV) statement for pharmacovigilance signal detection (15). Additionally, when selecting detection algorithms, we recognized the considerations and inherent limitations when using spontaneous reporting systems as a source of clinical data (16).

Sex differences analysis

To formally compare sex differences beyond descriptive counts, we conducted a between-sex comparative disproportionality analysis using the relative reporting odds ratio (rROR). For each drug–event pair (PT), we constructed a sex-stratified 2×2 table within reports of the target drug, where ${\text{a}}_{\text{m}}$ and ${\text{b}}_{\text{m}}$ denote the numbers of male reports with the PT and with other PTs, and ${\text{c}}_{\text{f}}$ and ${\text{d}}_{\text{f}}$ denote the corresponding counts in female reports. The sex contrast was quantified as:


$${\text{rROR}}_{m/f}=\frac{a_{m}/b_{m}}{c_{f}/d_{f}}=\frac{a_{m}d_{f}}{b_{m}c_{f}}.$$

Confidence intervals and hypothesis testing were performed on the log scale using the standard large-sample approximation for ln(rROR m/f), yielding 95% CIs and two-sided p-values. Multiple comparisons across PTs were adjusted using the Benjamini–Hochberg false discovery rate (BH-FDR).

An ${\text{rROR}}_{m/f}$>1 indicates stronger reporting disproportionality in males, whereas ${\text{rROR}}_{m/f}$<1 indicates stronger disproportionality in females.

In this study, four established disproportionality algorithms were applied for signal detection: the Reporting Odds Ratio (ROR) (17), Proportional Reporting Ratio (PRR) (18), Bayesian Confidence Propagation Neural Network (BCPNN) (19), and Multi-Item Gamma Poisson Shrinker (MGPS) method (20). Each algorithm offers distinct advantages: ROR helps correct biases related to low event counts; PRR provides higher specificity compared with ROR; BCPNN excels at integrating multisource data and performing cross-validation; and MGPS is particularly effective in identifying signals from rare events.

The mathematical formulations and significance thresholds for these four algorithms are summarized in **Supplementary Table 1**. All statistical analyses were performed using R software. Higher signal values indicate stronger associations between the target drug and specific ADEs, suggesting a greater likelihood of a causal relationship.

## 3 Results

### 3.1 Demographic information of adverse events for ALK-TKI drugs

As of the second quarter of 2025, the FAERS database received a total of 9,030 adverse event reports for Crizotinib, 8,486 for Alectinib, and 1,167 for Brigatinib.

For Crizotinib, the baseline data shows that the number of female patients slightly exceeds the number of male patients (48.9% vs. 39.9%). Among reports with clear age data, the most common age group was 18–65 years (40.4%). The majority of patients had a weight range between 50–100 kg (20.1%), though weight information was missing for 74.2% of the population. The predominant indication was LUNG NEOPLASM MALIGNANT (29.8%). The majority of reports came from the United States (44.8%), followed by India (11.6%) and Japan (7.8%).

For Alectinib, female patients were more prominent than male patients (54.6% vs. 36.5%). Among reports with clear age data, the most common age group was also 18–65 years (34.3%). The weight range for most patients was 50–100 kg (19.8%), with 76.8% missing weight data. The predominant indication was LUNG NEOPLASM MALIGNANT (31.9%). The majority of reports came from the United States (51.0%), followed by Japan (8.2%) and China (7.1%).

For Brigatinib, female patients slightly outnumbered male patients (49.4% vs. 40.7%). The most common age group among reports with clear age data was 18–65 years (24.0%). The majority of patients had a weight range between 50–100 kg (12.7%), with weight information missing for 84.7% of the population. The predominant indication was LUNG NEOPLASM MALIGNANT (30.4%). The majority of reports came from Japan (47.9%), followed by the United States (31.1%) and France (3.8%). Detailed information is provided in **Table 1**.

**Table 1:** Clinical characteristics of Crizotinib, Alectinib, and Brigatinib adverse event reports from the FAERS database (Storage time - Q2 2025).

Characteristics	Crizotinib		Alectinib		Brigatinib	
Number of events	9030		8486		1167	
Gender,number(%)						
Female	4417 (48.9%)		4636 (54.6%)		576 (49.4%)	
Male	3600 (39.9%)		3098 (36.5%)		475 (40.7%)	
Miss	1013 (11.2%)		752 (8.9%)		116 (9.9%)	
Age,number(%)						
Median Age	65.0		75.0		41.4	
<18	303 (3.4%)		249 (2.9%)		318 (27.2%)	
18-65	3646 (40.4%)		2907 (34.3%)		280 (24.0%)	
65-85	2799 (31.0%)		2082 (24.5%)		204 (17.5%)	
>85	/		/		6 (0.5%)	
Miss	2099 (23.2%)		3156 (37.2%)		359 (30.8%)	
Weight(KG),number(%)						
<50	391 (4.3%)		166 (2.0%)		18 (1.5%)	
50-100	1811 (20.1%)		1683 (19.8%)		148 (12.7%)	
≥100	132 (1.5%)		119 (1.4%)		13 (1.1%)	
Miss	6696 (74.2%)		6518 (76.8%)		988 (84.7%)	
Top 5 indication,number(%)	LUNG NEOPLASH MALIGHANT	(1844, 29.8%)	LUNG NEOPLASM MALIGNANT	(2713, 31.9%)	LUNG NEOPLASM MALIGNANT	355(30.4%)
	HOH-SHALL CELL LUHG CANCER	(1635, 20.4%)	NON-SMALL CELL LUNG CANCER	(1771, 20.8%)	NON-SMALL CELL LUNG CANCER	(323, 27.6%)
	LUHG ADEOCARCIHOMA	(404, 18.1%)	PRODUCT USED FOR UNKNOWN INDICATION	(1219, 14.3%)	PRODUCT USED FOR UNKNOWN INDICATION	(238, 20.4%)
	NON SMALL CELL LUNG CACER METASTATIC	366(4.4%)	NON-SMALL CELL LUNG CANCER METASTATIC	(583,6.8%)	LUNG ADENOCARCINOMA	(48,4.1%)
	NEOPLASM MALIGHANT	319(1.4%)	LUNG ADENOCARCINOMA	(451, 5.3%)	LUNG ADENOCARCINOMA STAGE IV	(32, 2.7%)
Top 5 Reported Countries,number(%)	UNITED STATES	4054(44.8%)	UNITED STATES	(4328, 51.0%)	JAPAN	(560, 47.9%)
	INDIA	1053(11.6%)	JAPAN	704(8.2%)	UNITED STATES	(364, 31.1%)
	JAPAN	712(7.8%)	CHINA	661(7.1%)	FRANCE	45, 3.8%)
	CHINA	498(5.5%)	ITALY	(248 , 2.9%)	KOREA	(22, 1.8%)
	FRANCE	363(4.0%)	FRANCE	(209,2.4%)	GERMANY	(14,1.1%)

**Figure 2:**
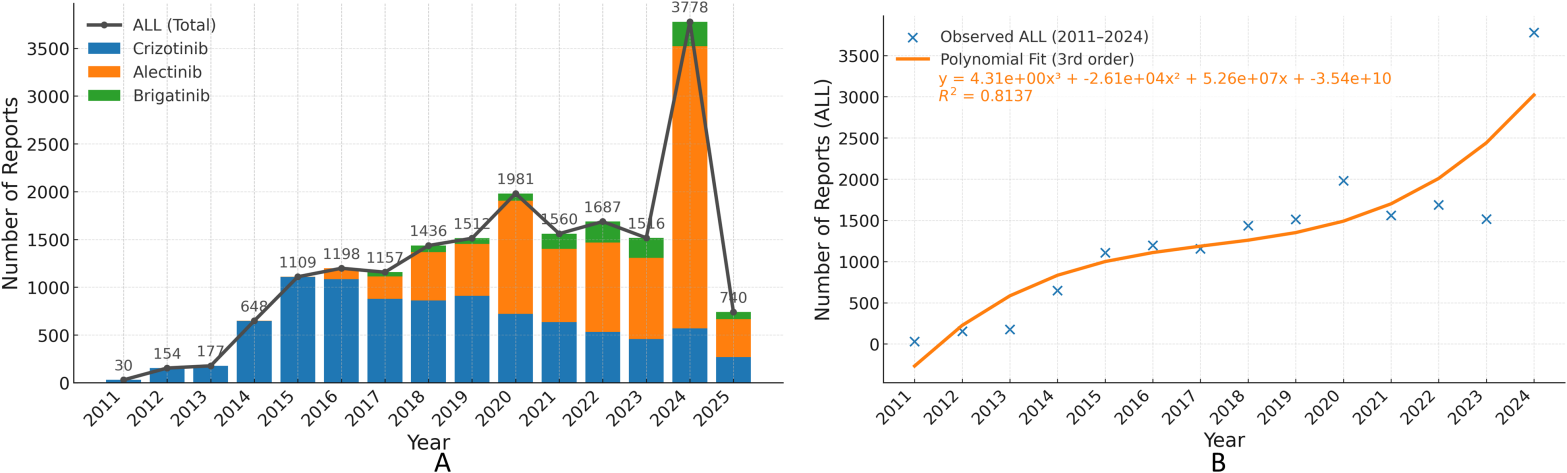
Reporting trends of adverse drug events (ADEs) associated with ALK-TKIs: crizotinib, alectinib, and brigatinib. **(A)** Bar chart of annual adverse event reports; **(B)** Polynomial fitting curve of annual adverse event reports.

**Figure 2A** shows that since 2011, Crizotinib, the first approved ALK-TKI, reached a preliminary peak of adverse event reports in 2015 (approximately 1,109 cases), followed by a gradual decline. Since 2016, reports for Alectinib have risen rapidly, surpassing Crizotinib in 2020 to become the leading contributor, peaking in 2024 with about 2,953 cases. Brigatinib began receiving reports in 2017, with relatively few reports overall, but a consistent increase year by year. Overall (ALL), the total number of adverse event reports peaked in 2024 (3,778 cases), indicating a significant increase in safety reports for ALK-TKI drugs as clinical applications expanded and their market presence grew.

Further polynomial curve fitting analysis of the total reports for the three drugs was conducted, as shown in **Figure 2B**. The adverse event reporting frequency for ALK-TKI drugs has shown a rapid growth trend. The coefficient of determination (R² = 0.8137) indicates that the model explains 81.37% of the data variability, which demonstrates a high level of reference value for this trend.

### 3.2 SOC-level signal analysis

**Figure 3:**
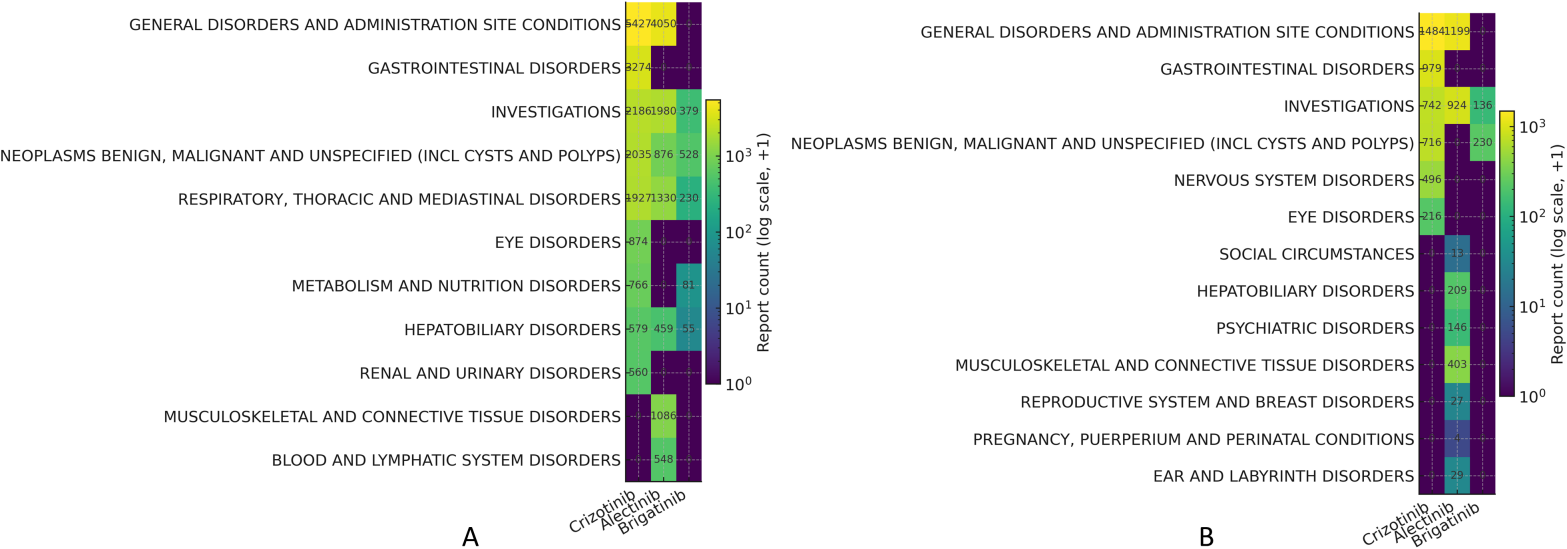
Heatmaps of ALK-TKI risk signals at the SOC level. **(A)** SOC-level risk signal heatmap in the overall population; **(B)** SOC-level risk signal heatmap in the lung cancer subgroup.

**Figure 3A** presents the System Organ Class (SOC)–level risk signals associated with ALK-TKIs. Among all reports, the most frequent SOC for crizotinib (N = 5,427) and alectinib (N = 4,050) was General disorders and administration site conditions, while Neoplasms benign, malignant and unspecified (including cysts and polyps) represented the predominant SOC for brigatinib (N = 528).

When restricting the analysis to cases with lung cancer as the reported indication (**Figure 3B**), the same pattern was observed: General disorders and administration site conditions remained the most frequent SOC for crizotinib (N = 1,484) and alectinib (N = 1,199), whereas Neoplasms benign, malignant and unspecified (including cysts and polyps) remained dominant for brigatinib (N = 230).

**Figure 4:**
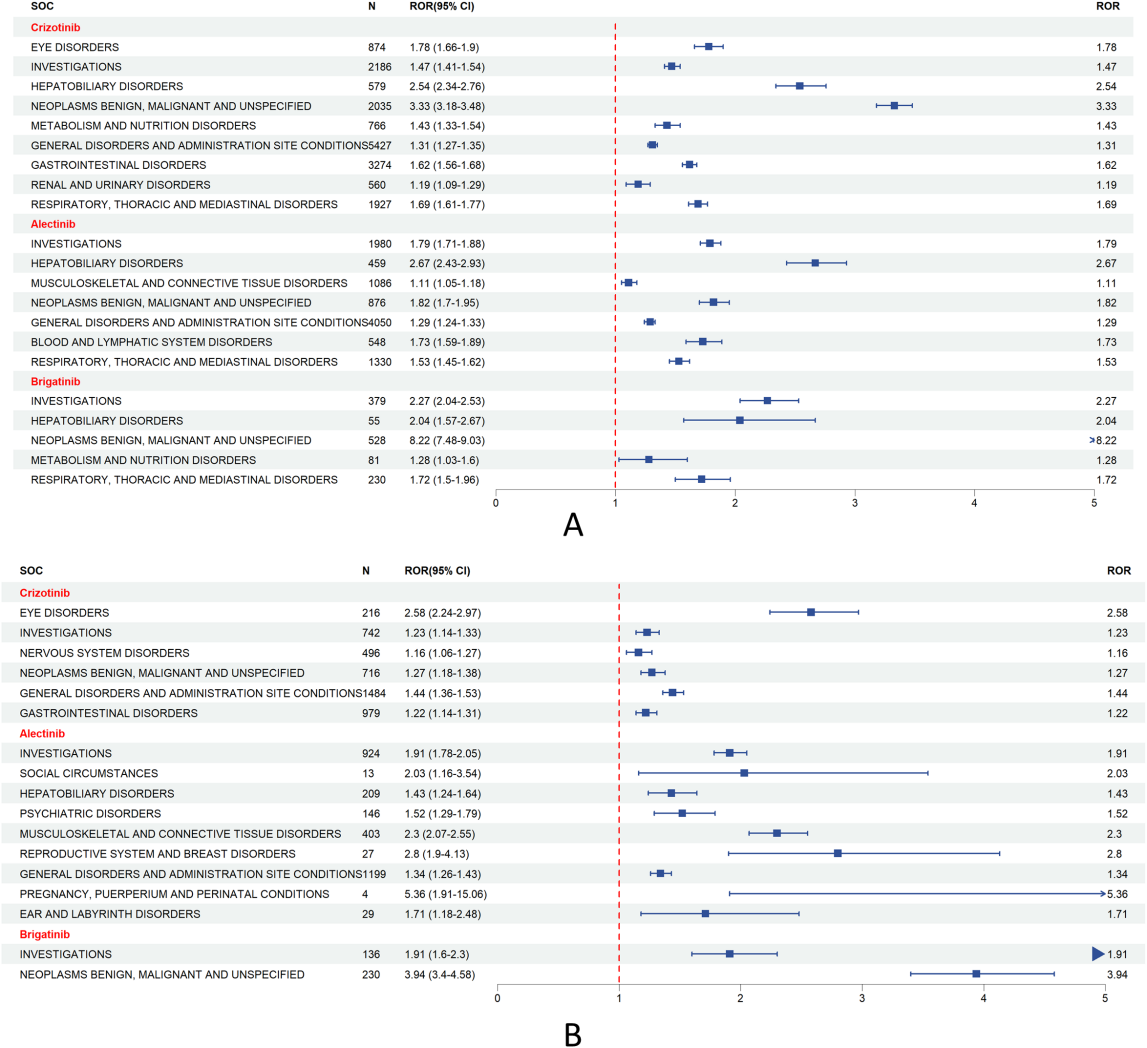
Forest plots of ALK-TKI risk signals at the SOC level. **(A)** SOC-level risk signal forest plot in the overall population; **(B)** SOC-level risk signal forest plot in the lung cancer subgroup.

A further assessment of SOC-level disproportionality signals is illustrated in the forest plots in **Figure 4**.

In the overall population (**Figure 4A**), the strongest risk signals were as follows: Crizotinib: Neoplasms benign, malignant and unspecified (ROR = 3.33, 95% CI = 3.18–3.48), Alectinib: Hepatobiliary disorders (ROR = 2.67, 95% CI = 2.43–2.93), Brigatinib: Neoplasms benign, malignant and unspecified (ROR = 8.22, 95% CI = 7.48–9.03), In the lung cancer subgroup (**Figure 4B**), the top risk signals were: Crizotinib: Eye disorders (ROR = 2.58, 95% CI = 2.24–2.97) Alectinib: Pregnancy, puerperium and perinatal conditions (ROR = 5.36, 95% CI = 1.91–15.06), Brigatinib: Neoplasms benign, malignant and unspecified (ROR = 3.94, 95% CI = 3.40–4.58).

### 3.3 PT-level signal analysis

**Figure 5:**
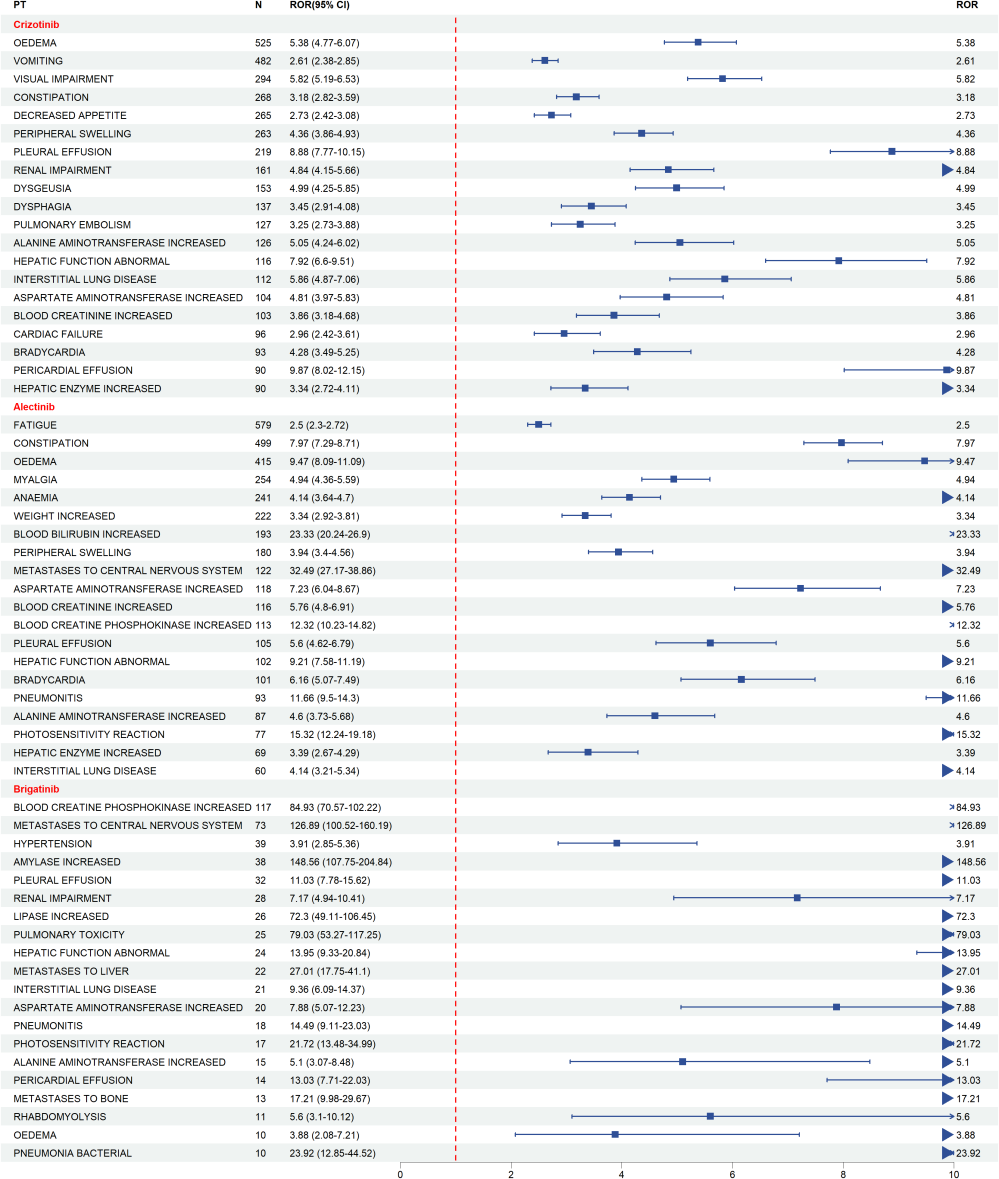
Forest plots of PT-level risk signals associated with ALK-TKIs.

**Figure 5** presents the forest plots of preferred term (PT)–level risk signals for the three ALK-TKIs. For crizotinib, the highest signals were observed for peripheral oedema (N = 525, ROR = 5.38, 95% CI = 4.77–6.07), vomiting (N = 482, ROR = 2.61, 95% CI = 2.38–2.85), and visual impairment (N = 294, ROR = 5.82, 95% CI = 5.19–6.53). For alectinib, strong signals were detected for fatigue (N = 579, ROR = 2.50, 95% CI = 2.30–2.72), constipation (N = 499, ROR = 7.97, 95% CI = 7.29–8.71), and oedema (N = 415, ROR = 9.47, 95% CI = 8.09–11.09). For brigatinib, similar risk signals were observed, with fatigue, constipation, and oedema showing relatively high ROR values.

**Figure 6:**
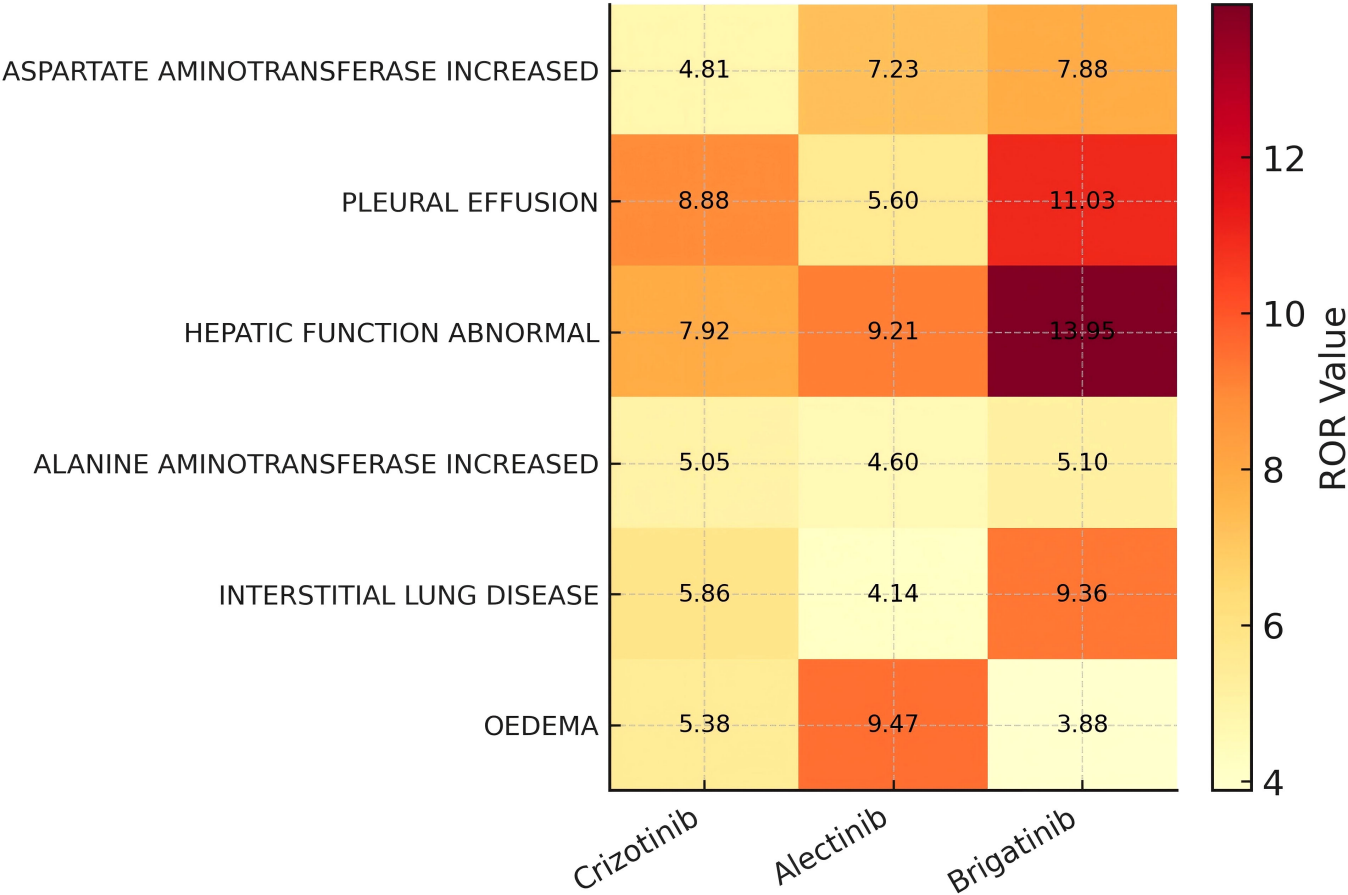
Heatmap of overlapping PT-level risk signals across ALK-TKIs.

To further compare overlapping adverse events across the three ALK-TKIs, six common PT-level risk signals were visualized in a heatmap (**Figure 6**) according to their ROR values. Overall, the three drugs shared partially overlapping ADE profiles, but the strength of signal varied among them, particularly for hepatic and pulmonary events, which consistently showed higher ROR values. Specifically, brigatinib demonstrated the strongest signals for hepatic function abnormal (ROR = 13.95), pleural effusion (ROR = 11.03), and interstitial lung disease (ROR = 9.36), indicating a higher risk of hepatotoxicity and pulmonary toxicity. Alectinib showed pronounced signals for oedema (ROR = 9.47) and hepatic function abnormal (ROR = 9.21), suggesting a tendency toward peripheral edema and hepatobiliary adverse effects. In contrast, crizotinib displayed a more balanced distribution of signals, with the highest RORs observed for pleural effusion (ROR = 8.88) and hepatic function abnormal (ROR = 7.92).

A between-sex comparative disproportionality analysis was subsequently conducted using the relative reporting odds ratio (rROR) (**Table 2**). For crizotinib, several PTs retained statistically robust sex contrasts after BH-FDR adjustment: female-enriched reporting was observed for nausea (rROR=0.623, 95% CI 0.528–0.734, p_FDR=3.49×10^−7^), vomiting (0.575, 0.471–0.702, p_FDR=4.96×10^−7^), malaise (0.673, 0.509–0.890, p_FDR=0.0277), and visual impairment (0.717, 0.555–0.925, p_FDR=0.0425). In contrast, renal impairment showed a male-enriched signal (1.905, 1.338–2.714, p_FDR=0.00235). Other PTs did not remain significant after multiplicity control.

**Table 2:** Between-sex comparative disproportionality analysis (relative reporting odds ratio, rROR) for three ALK-TKIs (Crizotinib, Alectinib, and Brigatinib) in FAERS.

Drug	PT	a_MALE	b_MALE	c_FEMALE	d_FEMALE	rROR_MvsF	95%CI	p(z)	p(FDR)
Crizotinib	NAUSEA	213	9716	459	13043	0.623	0.528–0.734	1.75E-08	0.000000349
Crizotinib	DIARRHOEA	192	9737	317	13185	0.82	0.684–0.983	0.032	0.106
Crizotinib	DYSPNOEA	142	9787	172	13330	1.124	0.899–1.406	0.304	0.695
Crizotinib	VOMITING	141	9788	330	13172	0.575	0.471–0.702	4.96E-08	0.000000496
Crizotinib	FATIGUE	126	9803	212	13290	0.806	0.645–1.006	0.057	0.162
Crizotinib	DECREASED APPETITE	116	9813	142	13360	1.112	0.869–1.423	0.398	0.695
Crizotinib	OEDEMA PERIPHERAL	115	9814	144	13358	1.087	0.853–1.386	0.5	0.695
Crizotinib	PNEUMONIA	109	9820	140	13362	1.061	0.825–1.364	0.646	0.837
Crizotinib	CONSTIPATION	105	9824	159	13343	0.897	0.700–1.149	0.39	0.695
Crizotinib	PERIPHERAL SWELLING	105	9824	155	13347	0.92	0.717–1.180	0.501	0.695
Crizotinib	ASTHENIA	104	9825	139	13363	1.017	0.787–1.313	0.945	0.999
Crizotinib	OFF LABEL USE	103	9826	130	13372	1.079	0.832–1.399	0.572	0.731
Crizotinib	DIZZINESS	93	9836	126	13376	1.002	0.764–1.313	0.999	0.999
Crizotinib	VISUAL IMPAIRMENT	91	9838	172	13330	0.717	0.555–0.925	0.0106	0.0425
Crizotinib	COUGH	89	9840	108	13394	1.123	0.839–1.504	0.435	0.695
Crizotinib	PLEURAL EFFUSION	84	9845	125	13377	0.914	0.682–1.225	0.548	0.731
Crizotinib	PYREXIA	79	9850	107	13395	1.004	0.737–1.368	0.999	0.999
Crizotinib	MALAISE	74	9855	149	13353	0.673	0.509–0.890	0.00555	0.0277
Crizotinib	RENAL IMPAIRMENT	74	9855	53	13449	1.905	1.338–2.714	0.000353	0.00235
Crizotinib	PRODUCT USE ISSUE	62	9867	75	13427	1.126	0.802–1.579	0.503	0.695
Alectinib	DISEASE PROGRESSION	136	6404	145	10709	1.568	1.239–1.986	0.000186	0.00371
Alectinib	WEIGHT INCREASED	56	6484	156	10698	0.592	0.436–0.805	0.000822	0.00822
Alectinib	BLOOD CREATININE INCREASED	53	6487	48	10806	1.839	1.243–2.721	0.0023	0.0129
Alectinib	DRUG INEFFECTIVE	98	6442	107	10747	1.528	1.160–2.013	0.00259	0.0129
Alectinib	ANAEMIA	63	6477	154	10700	0.676	0.503–0.907	0.00917	0.0336
Alectinib	CONSTIPATION	150	6390	320	10534	0.773	0.635–0.940	0.0101	0.0336
Alectinib	PNEUMONIA	80	6460	93	10761	1.433	1.061–1.935	0.0189	0.0508
Alectinib	ASTHENIA	101	6439	123	10731	1.368	1.050–1.784	0.0203	0.0508
Alectinib	BLOOD BILIRUBIN INCREASED	81	6459	96	10758	1.405	1.044–1.892	0.0249	0.0553
Alectinib	BRADYCARDIA	44	6496	47	10807	1.553	1.011–2.385	0.0445	0.0704
Alectinib	DIARRHOEA	51	6489	105	10749	0.804	0.578–1.120	0.196	0.267
Alectinib	FATIGUE	193	6347	342	10512	0.937	0.785–1.118	0.471	0.566
Alectinib	PERIPHERAL SWELLING	60	6480	117	10737	0.849	0.619–1.165	0.31	0.566
Alectinib	ARTHRALGIA	50	6490	71	10783	1.17	0.814–1.682	0.397	0.567
Alectinib	RASH	89	6451	166	10688	0.888	0.679–1.161	0.382	0.567
Alectinib	DYSPNOEA	101	6439	158	10696	1.061	0.822–1.369	0.65	0.742
Alectinib	NAUSEA	60	6480	113	10741	0.881	0.639–1.215	0.442	0.695
Alectinib	COUGH	55	6485	98	10756	0.931	0.660–1.315	0.686	0.762
Alectinib	MYALGIA	88	6452	141	10713	1.036	0.784–1.369	0.805	0.847
Alectinib	PAIN	56	6484	97	10757	0.957	0.677–1.352	0.806	0.847
Brigatinib	NAUSEA	20	1172	43	1498	0.594	0.348–1.016	0.0572	0.607
Brigatinib	METASTASES TO LIVER	13	1179	7	1534	2.416	0.961–6.075	0.0607	0.607
Brigatinib	BLOOD CREATINE PHOSPHOKINASE INCREASED	52	1140	55	1486	1.232	0.837–1.815	0.29	0.941
Brigatinib	PRODUCT DOSE OMISSION ISSUE	9	1183	16	1525	0.725	0.319–1.647	0.442	0.941
Brigatinib	DYSPNOEA	9	1183	15	1526	0.774	0.338–1.775	0.545	0.941
Brigatinib	PYREXIA	10	1182	17	1524	0.758	0.346–1.662	0.49	0.941
Brigatinib	RENAL IMPAIRMENT	11	1181	13	1528	1.095	0.489–2.452	0.826	0.941
Brigatinib	COUGH	11	1181	15	1526	0.948	0.434–2.071	0.893	0.941
Brigatinib	RASH	12	1180	23	1518	0.671	0.333–1.354	0.266	0.941
Brigatinib	PLEURAL EFFUSION	14	1178	17	1524	1.065	0.523–2.170	0.861	0.941
Brigatinib	DECREASED APPETITE	15	1177	15	1526	1.297	0.631–2.663	0.479	0.941
Brigatinib	AMYLASE INCREASED	15	1177	18	1523	1.078	0.541–2.149	0.83	0.941
Brigatinib	DRUG INEFFECTIVE	16	1176	24	1517	0.86	0.455–1.626	0.643	0.941
Brigatinib	HYPERTENSION	17	1175	19	1522	1.159	0.600–2.240	0.661	0.941
Brigatinib	FATIGUE	18	1174	17	1524	1.374	0.705–2.679	0.35	0.941
Brigatinib	METASTASES TO CENTRAL NERVOUS SYSTEM	29	1163	36	1505	1.042	0.635–1.710	0.869	0.941
Brigatinib	DIARRHOEA	30	1162	41	1500	0.945	0.586–1.522	0.815	0.941
Brigatinib	ALANINE AMINOTRANSFERASE INCREASED	8	1184	6	1535	1.729	0.598–4.996	0.312	0.941
Brigatinib	ASPARTATE AMINOTRANSFERASE INCREASED	8	1184	11	1530	0.94	0.377–2.344	0.894	0.941
Brigatinib	PULMONARY TOXICITY	11	1181	14	1527	1.016	0.460–2.246	0.969	0.969

1 PT indicates the MedDRA preferred term. 2 a_MALE and b_MALE are the numbers of male reports with the PT of interest and with other PTs (non-PT) within the same drug, respectively; c_FEMALE and d_FEMALE are the corresponding counts in female reports. 3 rROR_MvsF compares disproportionality between males and females: values >1 indicate stronger reporting disproportionality in males, whereas values<1 indicate stronger disproportionality in females.4 95%CI denotes the 95% confidence interval for rROR; p(z) denotes the two-sided p-value from the log-scale z-test. 5 p(FDR) denotes Benjamini–Hochberg false discovery rate–adjusted p-values, calculated within each drug across the PTs listed for that drug.

For alectinib, rROR analysis indicated male-enriched reporting for disease progression (1.568, 1.239–1.986, p_FDR=0.00371), drug ineffective (1.528, 1.160–2.013, p_FDR=0.0129), and blood creatinine increased (1.839, 1.243–2.721, p_FDR=0.0129), whereas female-enriched reporting was identified for weight increased (0.592, 0.436–0.805, p_FDR=0.00822), constipation (0.773, 0.635–0.940, p_FDR=0.0336), and anaemia (0.676, 0.503–0.907, p_FDR=0.0336). Signals such as pneumonia and asthenia showed borderline significance after correction (both p_FDR≈0.0508).

For brigatinib, no PT-level sex contrast remained statistically significant following BH-FDR adjustment (all p_FDR≥0.607). Although nausea tended to be female-enriched (rROR=0.594, 0.348–1.016, p=0.0572) and metastases to liver tended to be male-enriched (2.416, 0.961–6.075, p=0.0607), these differences did not withstand multiplicity correction, likely reflecting limited statistical power.

Collectively, the between-sex comparative results demonstrate drug-specific sex contrasts in reporting disproportionality, with the most consistent differences observed for crizotinib and alectinib, whereas brigatinib exhibited no robust sex-associated variation in the PTs analyzed.

### 3.4 External validation using the Canadian CVARDD database

To further ensure the accuracy and robustness of the findings, an external validation analysis was conducted using adverse event data from the Canadian Vigilance Adverse Reaction Database (CVARDD).

**Figure 7:**
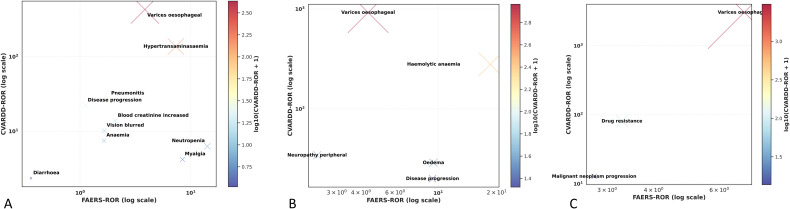
External validation of ALK-TKI PT-level ROR signals in the CVARDD database. **(A)** Comparison of crizotinib ROR values between FAERS and CVARDD (bubble chart); **(B)** Comparison of alectinib ROR values between FAERS and CVARDD (bubble chart); **(C)** Comparison of brigatinib ROR values between FAERS and CVARDD (bubble chart).

**Figure 7A** compares the preferred term (PT)–level ROR signals of crizotinib between the FAERS and CVARDD databases to assess the external consistency of signal detection. Overall, most adverse events exhibited similar signal trends across both datasets, indicating good reproducibility of the results. Notably, oesophageal varices (ROR = 429.45) and hypertransaminasaemia (ROR = 136.98) showed substantially stronger signals in CVARDD than in FAERS, suggesting that despite differences in population composition and reporting structure between the two databases, the direction of risk association remained consistent. In contrast, common adverse events such as diarrhoea and anaemia displayed moderate and stable signal intensities in both datasets, reflecting the reliability of these associations. On a logarithmic scale, the signal distributions and color gradients showed a strong positive correlation between FAERS and CVARDD, further confirming the cross-database consistency and external robustness of crizotinib-related risk signals.

**Figure 7B** presents the comparison of PT-level ROR signals for alectinib across the two databases. In CVARDD, oesophageal varices (ROR = 921.40) and haemolytic anaemia (ROR = 279.11) exhibited markedly higher signal intensities than those observed in FAERS, implying potential amplification effects in real-world clinical data while maintaining consistent directional associations. Meanwhile, oedema and disease progression demonstrated moderate and directionally concordant signals in both databases, reinforcing the stability and reliability of alectinib-related adverse events. The overall positive correlation between signal distributions across databases further supports the robustness and external validity of alectinib-associated safety signals.

As shown in **Figures 7C**, the PT-level signal analysis for brigatinib revealed that oesophageal varices exhibited an exceptionally strong signal in the CVARDD database (ROR = 3,042.52), significantly higher than its corresponding FAERS value (ROR = 7.01). This finding indicates a pronounced signal amplification effect in real-world clinical settings, although the association direction remained consistent. Additionally, drug resistance (ROR = 80.72) and malignant neoplasm progression (ROR = 12.74) both showed concordant positive signals across the two databases, further corroborating the reliability of these associations.

Taken together, all three ALK-TKIs—crizotinib, alectinib, and brigatinib—demonstrated consistent cross-database risk signal trends between FAERS and CVARDD, underscoring the high external robustness and reproducibility of this pharmacovigilance analysis.

### 3.5 Time-to-onset analysis of safety signals

In this study, the temporal characteristics of adverse events (AEs) were evaluated using median time-to-onset (TTO) and Weibull distribution modeling. The Weibull model provides two key parameters—shape (β) and scale (α)—to describe the temporal risk pattern of AEs. When the shape parameter β< 1 and its 95% CI< 1, the incidence of AEs is considered to decrease over time, representing an “early failure” pattern. When β ≈ 1 and the 95% CI includes 1, AEs occur randomly over time, indicating a “random failure” pattern.

**Figure 8:**
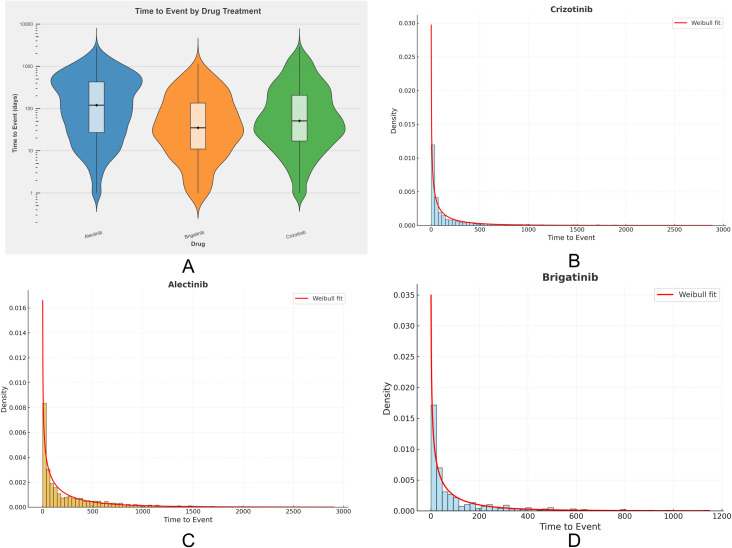
Time-to-onset analysis of adverse events associated with ALK-TKIs. **(A)** Violin plot of time-to-onset distributions for ALK-TKI–related adverse events; **(B–D)** Weibull distribution curves of time-to-onset for crizotinib, alectinib, and brigatinib, respectively.

As summarized in **Table 3**, a total of 2,348 valid reports for crizotinib, 2,211 for alectinib, and 424 for brigatinib included time-to-onset information. The median TTOs were 51 days, 120 days, and 35 days, respectively. All three drugs exhibited an early failure pattern (β< 1), suggesting that the risk of ADEs was highest during the initial treatment phase and declined thereafter. The corresponding Weibull distribution curves for each drug are shown in **Figures 8B–D**.

**Table 3:** Time to onset of Crizotinib, Alectinib, and Brigatinib adverse events and Weibull distribution analysis.

Drug	TTO(days)		Weibull distribution		
	Case reports	Median(day)	Scale parameter: α(95%CI)	Shape parameter: β(95%CI)	Type
Crizotinib	2348	51	137.58(119.58~128.58)	0.63(0.59~0.61)	Early failure
Alectinib	2211	120	242.19(212.74~227.47)	0.70(0.66~0.68)	Early failure
Brigatinib	424	35	94.31(69.05~81.68)	0.70(0.60~0.65)	Early failure
Crizotinib(HEPATIC FUNCTION ABNORMAL)	54	14.5	40.11(16.20~28.16)	0.79(0.55~0.67)	Early failure
Alectinib(HEPATIC FUNCTION ABNORMAL)	43	34	183.05(55.75~119.40)	0.73(0.46~0.59)	Early failure
Brigatinib(HEPATIC FUNCTION ABNORMAL)	17	19	59.95(7.23~33.59)	0.85(0.43~0.64)	Early failure
Crizotinib(PLEURAL EFFUSION)	80	34.5	130.28(53.65~91.67)	0.65(0.47~0.56)	Early failure
Alectinib(PLEURAL EFFUSION)	28	51.5	179.63(48.20~113.92)	0.86(0.50~0.68)	Early failure
Brigatinib(PLEURAL EFFUSION)	16	55.5	75.05(31.44~53.24)	1.77(0.71~1.24)	Random Failure Curve

TTO, time to onset; CI, confidence interval.

A violin plot of TTO distributions across the three drugs (**Figure 8A**) further illustrates inter-drug variability. Crizotinib displayed the widest temporal dispersion and the broadest range of AE onset times, reflecting greater interindividual differences in its safety profile.

We further performed TTO analyses for two clinically important high-signal AEs—hepatic function abnormal and pleural effusion—as detailed in **Table 3**. For hepatic function abnormal, brigatinib showed the shortest median TTO (19 days), with all three drugs following early failure–type Weibull curves, indicating that liver injury tends to occur early in treatment. For pleural effusion, crizotinib demonstrated the shortest median TTO (34.5 days). Both crizotinib and alectinib exhibited early failure patterns, whereas brigatinib displayed a random failure–type curve, suggesting continuous risk of pleural effusion over time.

## 4 Discussion

This study conducted a systematic comparison of adverse drug event (ADE) signals associated with three representative anaplastic lymphoma kinase tyrosine kinase inhibitors (ALK-TKIs)—crizotinib, alectinib, and brigatinib—using two large global real-world pharmacovigilance databases: FAERS and CVARDD. The analysis focused on differences in adverse event profiles, sex-related variations, and time-to-onset characteristics (21). The results revealed significant heterogeneity in the safety profiles of these ALK-TKIs, which appear to be influenced by drug generation, structural characteristics, metabolic pathways, and individual patient factors. The findings provide real-world evidence to support clinical medication management and individualized risk control in patients with ALK-positive non–small cell lung cancer (NSCLC).

### 4.1 Adverse event characteristics and population differences

The results of the study indicate that all three ALK-TKIs primarily present with systemic disorders and administration site reactions at the System Organ Class (SOC) level. At the specific adverse event level, liver function abnormalities, edema, fatigue, pleural effusion, and visual disturbances are the most common types of adverse reactions. There are significant differences in the adverse event profiles of the different drugs: Brigatinib exhibits the strongest signals for liver injury and pulmonary toxicity (liver dysfunction ROR = 13.95, interstitial lung disease ROR = 9.36), suggesting that this drug should be used with caution in patients with impaired liver function or underlying pulmonary diseases (12, 22, 23); Alectinib’s major adverse events are concentrated in metabolic and fluid retention reactions (e.g., edema ROR = 9.47), which could exacerbate circulatory burden in patients with heart failure, renal insufficiency, or prolonged sedentary behavior (24, 25); Crizotinib’s adverse event distribution is relatively balanced, but the risks of visual disturbances and pleural effusion are higher (visual disturbances ROR = 5.82, pleural effusion ROR = 8.88), so monitoring should be strengthened for patients who drive or perform fine tasks, as well as for those with pleural diseases.

In recent years, according to a national analysis from Greece, the usage rate of Crizotinib has significantly decreased, while Alectinib’s usage has notably increased. From 2020 to 2022, the usage of Alectinib rose from 16% to 59%, reflecting the widespread adoption of next-generation ALK inhibitors in clinical practice (26). This trend indicates that Alectinib, along with other new-generation ALK-TKIs, is gradually establishing its dominant position in treatment, especially in patients who require better control of brain metastases and superior resistance, gradually replacing Crizotinib as the standard treatment option.

Age distribution results show that patients aged 18–65 years are the main affected group, often in long-term treatment stages, where prolonged drug exposure increases the likelihood of cumulative toxicity (27, 28). Elderly patients, due to reduced liver and kidney metabolism capacity, should have their dosage appropriately reduced at the beginning of treatment, with shorter monitoring intervals.

Geographic distribution analysis shows that the United States and Japan have the highest number of reports, suggesting potential specific metabolic differences in East Asian populations. Previous studies have shown that the CYP3A5 genotype distribution in Asian patients differs from that in Western populations, potentially leading to higher plasma concentrations of Alectinib and Brigatinib, which increases the risk of hepatic and metabolic adverse reactions. Therefore, it is recommended to adopt a conservative dose escalation strategy in Asian patients, with adjustments to the dosing regimen based on plasma drug concentration monitoring (29).

In summary, for patients with liver dysfunction or chronic liver disease, Alectinib can be considered the first-choice drug, while Brigatinib should be used with caution. For patients with heart failure or renal impairment (30), Crizotinib offers relatively better safety. For younger patients requiring long-term maintenance treatment, Alectinib shows more significant overall tolerability and quality of life improvement advantages (31).

### 4.2 Sex differences and drug-specific risk profiles

Using the between-sex comparative disproportionality approach (rROR with BH-FDR adjustment), we observed drug-specific and PT-specific sex contrasts rather than uniform sex effects across all ALK-TKIs. In the crizotinib group, female-enriched reporting was identified for nausea, vomiting, malaise, and visual impairment (all rROR<1; BH-FDR<0.05), whereas renal impairment showed a male-enriched signal (rROR>1; BH-FDR<0.01). These findings suggest that sex may modulate the clinical expression of crizotinib-related toxicities, with males showing a stronger propensity for renal-related events and females showing relatively higher reporting of gastrointestinal and visual symptoms. Potential explanations include sex-related differences in body composition and pharmacokinetic handling (e.g., distribution volume and clearance), as well as hormonal influences on renal hemodynamics and symptom perception thresholds (32).

In the alectinib group, rROR analysis indicated male-enriched reporting for disease progression, drug ineffective, and blood creatinine increased (rROR>1; BH-FDR<0.05), whereas female-enriched reporting was observed for weight increased, constipation, and anaemia (rROR<1; BH-FDR<0.05). The sex contrasts for laboratory- and symptom-based events (e.g., creatinine increase, anaemia, constipation) may reflect sex-related variability in exposure and organ vulnerability (33, 34). Notably, the presence of sex differences in “disease progression” and “drug ineffective” should be interpreted cautiously, as these terms may capture complex real-world clinical contexts (e.g., treatment line, disease burden, or reporting practices) rather than direct biological susceptibility alone.

For brigatinib, no PT-level sex contrast remained statistically significant after BH-FDR adjustment, suggesting comparatively weaker or less detectable sex-associated variation in the PTs analyzed. Although nausea tended to be female-enriched and metastases to liver tended to be male-enriched at nominal significance, these differences did not withstand multiplicity control, likely reflecting limited statistical power and smaller report counts (35).

Overall, the results indicate that sex-related differences in ALK-TKI safety are agent-specific and endpoint-specific, and that rROR-based between-sex comparisons with multiplicity control help distinguish robust contrasts from descriptive fluctuations. Clinically, these findings support sex-informed, toxicity-targeted monitoring rather than broad sex-based assumptions. For example, early attention to renal-related events may be particularly relevant in males treated with crizotinib, whereas proactive assessment of gastrointestinal symptoms and visual complaints may be more pertinent in females. For alectinib, monitoring priorities may differ by sex, with consideration of creatinine changes and effectiveness-related reports in males and vigilance for constipation, weight increase, and anaemia in females (35). Importantly, these implications should be interpreted as risk-stratification cues derived from pharmacovigilance signals; treatment modifications should follow established clinical guidelines and individual patient context.

### 4.3 Time-to-onset characteristics and risk management

The time-to-onset (TTO) analysis based on Weibull modeling indicated an early-failure pattern (β< 1) for the three ALK-TKIs, suggesting that the hazard of AE occurrence is relatively higher shortly after treatment initiation and decreases over time. Consistently, the median TTOs for crizotinib, alectinib, and brigatinib were 51, 120, and 35 days, respectively, indicating that AE onset is generally concentrated in the early treatment phase, although the distribution differs across agents (with a relatively longer onset profile observed for alectinib).

At the PT level, hepatic function abnormal and pleural effusion were among the earliest events (median 14.5–55 days). Notably, brigatinib showed a short median onset for hepatotoxicity (19 days), consistent with an early and more predictable toxicity profile that may be amenable to proactive surveillance. These observations are supported by published case reports. For example, reversible hepatic dysfunction has been reported during crizotinib therapy and resolved after appropriate management (36). In addition, contralateral pleural effusion has been described within days after crizotinib initiation and improved after drug discontinuation (37). Together, these reports provide clinical context for the early-onset tendency of selected hepatic and pulmonary AEs associated with ALK-TKIs.

From a risk-management perspective, the early-failure onset pattern implies that enhanced monitoring should be prioritized soon after treatment initiation, particularly for organ systems implicated by early-onset signals. Clinicians may consider closer follow-up during the early treatment period (e.g., the first 8–12 weeks), including targeted assessment of liver function and evaluation of respiratory symptoms when indicated. Importantly, TTO patterns should be interpreted as supporting the timing of vigilance rather than prescribing uniform schedules, and management decisions should follow established clinical guidelines and individual patient risk profiles.

### 4.4 Limitations

External validation using the CVARDD database confirmed the robustness of FAERS-derived signal detection results. The three ALK-TKIs demonstrated consistent directional trends across both databases, particularly for hepatic function abnormal, anaemia, and oesophageal varices, supporting the reliability of cross-database pharmacovigilance analysis. Nonetheless, several limitations must be acknowledged.

First, both FAERS and CVARDD are spontaneous reporting systems and are therefore subject to reporting bias: severe AEs are more likely to be reported, whereas mild, self-limiting, or expected events may be underreported. Second, incomplete clinical information (e.g., body weight, dosing details, treatment line, and concomitant medications) limited more granular analyses, including adjustment for confounding and exploration of dose–response relationships. Third, in accordance with the READER-PV guidelines for pharmacovigilance studies, several inherent constraints of spontaneous reporting data must be explicitly recognized. These databases lack a valid denominator of exposed patients; thus, true AE incidence rates cannot be estimated. In addition, key background variables—baseline comorbidities, prior anticancer therapies, exact dosing regimens, and molecular profiles—are frequently missing, restricting subgroup stratification and limiting causal interpretation.

Importantly, disproportionality-based methods identify statistical associations rather than causality. Therefore, the observed sex contrasts and time-to-onset patterns should be interpreted as hypothesis-generating signals that inform risk stratification and monitoring priorities. Future research should aim to (i) validate key signals using clinically annotated real-world datasets (e.g., electronic health records or claims-linked registries) that enable adjustment for treatment line, disease burden, comorbidities, and concomitant drugs; (ii) conduct targeted subgroup analyses (e.g., age strata, CNS metastasis status, renal/hepatic dysfunction at baseline, and concomitant cardiovascular medications) to clarify effect modification; (iii) apply longitudinal designs with longer follow-up to evaluate persistence, recurrence, and outcomes of major toxicities; and (iv) integrate pharmacokinetic/pharmacogenomic or pharmacometabolomic evidence to elucidate mechanisms underlying sex-related and early-onset toxicities.

## 5 Conclusion

In summary, this study systematically characterized differences among three ALK-TKIs in terms of adverse event profiles, sex-specific variations, and time-to-onset patterns. All three drugs predominantly induced hepatobiliary and metabolic toxicities, with brigatinib presenting the highest risk of early hepatotoxicity, alectinib showing a greater metabolic burden, and crizotinib demonstrating an overall balanced safety profile.

Sex-related differences were most pronounced for cardiac–renal and hepatic–hematologic AEs, underscoring the necessity of implementing sex-specific pharmacovigilance strategies. Furthermore, the “early failure” distribution pattern observed across all agents highlights the need for intensive early-phase monitoring and timely intervention.

These findings provide real-world evidence to support personalized safety management for ALK-TKI therapy and lay the groundwork for future sex-specific drug safety research and precision risk stratification model development in the context of targeted cancer therapies.

## Data Availability

The original contributions presented in the study are included in the article/**Supplementary Material**. Further inquiries can be directed to the corresponding author.
